# ‘They’re Going to Smoke Anyway’: A Qualitative Study of Community Mental Health Staff and Consumer Perspectives on the Role of Social and Living Environments in Tobacco Use and Cessation

**DOI:** 10.3389/fpsyt.2019.00503

**Published:** 2019-07-17

**Authors:** Laura Twyman, Carla Cowles, Scott C. Walsberger, Amanda L. Baker, Billie Bonevski, the Tackling Tobacco Mental Health Advisory Group, Karina Ko

**Affiliations:** ^1^Tabacco Control Unit, Cancer Council NSW, Woolloomooloo, NSW, Australia; ^2^School of Medicine and Public Health, Faculty of Health and Medicine, University of Newcastle, Callaghan, NSW, Australia; ^3^Human Capital Alliance, Potts Point, NSW, Australia

**Keywords:** community mental health, tobacco, mental illness, housing, living environment, social networks, qualitative

## Abstract

**Background:** Addressing the high prevalence of tobacco use experienced by people with severe mental illness (SMI) requires consideration of the influence of wider cultural, socioeconomic and environmental factors. This qualitative study aimed to examine the impact of social and living environments on tobacco use and cessation by people with SMI accessing community managed mental health services. The perspectives of both staff and consumers with SMI were explored.

**Methods:** Semi-structured focus groups were undertaken with a purposive sample of community mental health staff and consumers from three sites in three major cities in NSW, Australia. Two sites provided outreach support, and one site provided residential support. Data were collected (2017–2018) until saturation was reached. Focus groups were audio-recorded and transcribed, and thematic analysis was conducted.

**Results:** Thirty-one staff and 17 consumers participated separately in six focus groups. Themes identified by staff included a degree of fatalism, conceptualising tobacco use as choice rather than addiction and tensions between cessation support and broader models of care. Staff viewed smoke-free home and mental health service policies as effective at promoting quitting but contradictory to recovery-oriented models of care. Consumers identified smoking as an integral part of life and social networks, as a way of maintaining control and lack of social support to quit as key themes. While many consumers reported smoking inside the home, others described enforcing smoke-free rules.

**Conclusion:** Social and living environments played an integral role in tobacco use and cessation for both staff and consumers. The role of community managed mental health organisations in addressing tobacco use within social and living environments was not strongly supported by staff and sometimes seen as antithetical to recovery-oriented models of care. Potential ways to address this include education and training for prospective and current community mental health organisation staff highlighting the synergy between the recovery-oriented model and provision of preventive health support.

## Introduction

The prevalence of tobacco use in Australia reached a historic low of ∼13% in 2016 (AIHW 2017); however, prevalence is disproportionately higher amongst people experiencing mental illness. Prevalence varies with symptom severity and mental health disorder. For example, in Australia, tobacco use is consistently higher for people experiencing psychological distress (22%) (1); people who have ever been diagnosed or treated for a mental health condition (29%) (1) and people living with anxiety disorders (33%) (2), affective disorders (43%) (2) and psychotic disorders (67%) (3).

There is evidence that reductions in smoking prevalence seen in the general population have not occurred in groups with severe mental illness (SMI) and that rates have remained relatively stable among people with psychotic disorders (1). There are numerous definitions of severe (or serious) mental illness. Definitions tend to include reference to specific disorders, the severity of symptoms and the extent to which they impact on a person’s functioning. In the current study, we use SMI to refer to diagnosed mental disorders including schizophrenia and other psychoses, bipolar disorder, severe anxiety and depression that result in functional impairment which substantially limits one or more major life activities (4).

Tobacco use is a leading risk factor for cancer, cardiovascular disease and respiratory disease. The disproportionate use of tobacco by people with SMI contributes to the gap in mortality experienced in this group (5). The financial and social harms of tobacco use are also exacerbated within this group (6). Tobacco use often increases the financial stress and social stigma felt by people with SMI. Studies have estimated that people with SMI spend approximately 27% of their income on tobacco (7, 8). There are numerous reasons for the disproportionate use of tobacco by people with SMI. These include genetic, individual, interpersonal, community, social and environmental factors. Shared genetic predispositions to both mental illness and tobacco, smoking to manage stress, mental health symptoms and medication side effects, the historic influence of institutionalisation and use of tobacco to control and reward behaviour, normalisation of tobacco and use of tobacco to combat boredom and social isolation have been documented (6, 9, 10). Additionally, documentation acquired reveals that the tobacco industry actively targets and markets to people with SMI and the organisations that provide mental health support (11, 12).

While smokers with SMI have higher levels of nicotine dependence (13, 14) and may require additional support to quit compared to people without SMI, smoking cessation is possible among people with SMI. Service providers’ lack of knowledge and skills and negative attitudes towards addressing smoking (15–17) and systemic barriers within mental healthcare settings (18, 19) prevent people with SMI from receiving optimal smoking cessation support. Beliefs that people with SMI are not interested in quitting smoking and that quitting may jeopardise a person’s mental health are commonly reported misperceptions held by health and other professionals (16, 17). However, evidence demonstrates that people with SMI are just as likely to express motivation and desire to quit smoking as the general population (20, 21). Furthermore, quitting smoking is not associated with increased depression, anxiety or stress (22).

There is a critical need to improve on the way tobacco is addressed with people with SMI (10, 23). Successfully addressing tobacco use in people with SMI requires examination of the wider social and environmental context (24). Socio-ecological models can help increase understanding of the factors within living and social environments of people with SMI that impact on tobacco use. Community managed mental health organisations (hereafter referred to as community mental health organisations) provide a large portion of care to people with SMI in Australia. In 2015–2016, 9.4 million service contacts were provided to approximately 410,000 people (25). In the same year, the most common principal diagnosis of people receiving care in these settings was schizophrenia, followed by depressive episode and bipolar affective disorder. In Australia, mental health settings in general are increasingly providing care through recovery-oriented models (26). Recovery-oriented models prioritise the lived experience of the consumer, challenge traditional notions of expertise and power differentials between staff and consumers and support consumers to define recovery through their own goals, wishes and aspirations (27). Definitions of recovery are not limited to ameliorating symptoms and instead are developed by individuals with influence from social processes. Community mental health organisations are well positioned to address tobacco with people who access their services, providing psychosocial support in a trusted setting. Using qualitative methodology, the current research study sought to gain an in-depth understanding of the ways in which living and social environments shape tobacco use and cessation within these settings from the perspective of both staff and consumers.

## Methods

### Study Design

The aim of qualitative research is to gain in-depth understanding of real-world problems. In contrast to quantitative research, generalizability is not a guiding principle of qualitative research (28–31). Semi-structured focus groups were conducted separately with staff and consumers of community mental health organisations from July 2017 to February 2018. Focus groups were chosen because they enable group discussion with members stimulating each other in sharing experiences and views and the potential for individual reflection in the context of hearing others’ views (31). This study was carried out in accordance with the recommendations of the National Statement on Ethical Conduct in Human Research. The Cancer Council NSW Human Research Ethics Committee (#306) approved the study protocol, and all subjects gave written informed consent in accordance with the Declaration of Helsinki.

### Setting

This qualitative study was conducted in community mental health organisations in NSW, Australia. Community mental health organisations provide support through government-funded programs that are underpinned by an integrated care and support model (32). These programs involve partnerships between non-government organisations specialising in mental health who provide psychosocial support and NSW government health teams who provide clinical support. Services provided by the community mental health services include employment and education, leisure and recreation, family and carer support, helpline and counselling services, accommodation support and outreach and promotion, information and advocacy. The provision of smoking cessation services in this sector varies across and within organisations. Most support is provided *via* outreach; however, a small proportion of services also provide residential support. Community mental health organisations primarily support people with SMI (32), such as schizophrenia, bipolar disorder or schizoaffective disorder.

### Sampling

Sites were eligible to participate in these focus groups if they provided community-based psychosocial support to people with SMI (including outreach and residential support). Purposeful sampling was used to attempt to include sites from both metropolitan and regional areas. Senior management of community mental health organisations provided permission for their organisation to participate in the qualitative research. Once organisational consent was provided, the research team worked with management to identify individual sites to participate. Site-specific managers and team leaders were briefed on the study aims and methods and asked to support recruitment of consumers for focus groups. Consumers were eligible to participate in the focus groups if they were currently engaged with the service, were either current smokers or ex-smokers or lived with a current smoker, aged 16 years or older and able to provide informed consent. Ability to provide informed consent was defined as ability to understand the study’s purpose, risk and benefits as detailed in the information sheet (33). Staff at sites assessed consumer eligibility. Staff were eligible to participate in the study if they were currently providing support to consumers at the site. Staff could participate regardless of their own smoking status.

### Procedure

Six focus groups were conducted across three sites. All focus groups were conducted by CC, a research consultant with a background in science communication and extensive experience in qualitative health research, tobacco control and the community mental health sector. A second consultant with a background in qualitative health research and health workforce planning attended two focus groups. All focus groups were conducted at participating sites in private meeting rooms. Focus groups were conducted separately for staff and consumers. Staff and consumer participants were informed that they could elect to complete one-on-one interviews if they preferred; however, none took up this offer. Participants were provided with an information sheet and consent form and were given the opportunity to ask questions about the research study before the group began. Short surveys were conducted with both consumers and staff prior to commencement of the focus group. Surveys for staff assessed age, gender, Aboriginal and/or Torres Strait Islander status, smoking status and role in the organisation. Surveys for consumers were identical except where ‘role’ was replaced with ‘current mental health diagnoses’. Consumers were provided with a $50 grocery card gift voucher for participating. Staff did not receive reimbursement for participating. Data were collected until saturation was reached (i.e., no new themes were occurring in either staff or consumer groups).

### Discussion Guide

Semi-structured discussion guides were tailored to staff and consumer focus groups. The questions were developed by the project team based on the research aims. Discussion guides for staff and consumers covered the following topics: smoking history and current smoking behaviour; smoke-free environments (community mental health organisations and consumer living environments); role of community mental health organisations in providing cessation support and the enablers and barriers to cessation.

### Analysis

Data were collected, transcribed and analysed once all focus groups had been completed. Transcripts were analysed using thematic analysis. Summary notes of observations and impressions were developed by CC after each focus group. Data were continuously reviewed and compared to identify similarities and differences between sites, participant groups and responses to specific questions. Questions were modified for subsequent focus groups.

Transcription was undertaken by CC once all focus groups were completed, allowing for immersion in the data. Each transcript was reviewed by CC to note initial impressions and understanding of the data. Impressions and initial emerging themes were discussed by the researchers. This process was used to develop an initial set of codes. The transcripts were then re-read and coded by CC for relevant or meaningful phrases and sections of the transcripts, such as themes or comments that were repeated by several participants. Codes were modified and revised as required to best represent the data and then arranged according to emerging themes. Final themes were reviewed and discussed with the second research consultant and the study authors to confirm accuracy of interpretation of the data.

### Trustworthiness (Validity)

A vital part of qualitative methodology is the reporting of strategies to ensure the rigour of the qualitative work (34). The current study employed many of these strategies. During this study, the researchers considered how their professional background, experience and prior assumptions (as public health and behavioural science researchers) would impact on data collection and the ability to facilitate open and honest responses from participants. This included being sensitive to the different values and priorities that mental health staff may have around addressing tobacco compared to tobacco control public health researchers. During analysis and synthesis, an attempt was made to ensure that data were not presented as being representative of all consumers and/or staff. Rather, information was analysed and reported by comparing similarities and differences between, within and across groups. Where relevant, a majority view was reported. However, it was equally important to acknowledge individual experience and perspectives. A reflexive approach was adopted for all stages of the research process. Researchers would summarise, reflect and feed back information to confirm or clarify data collected within focus groups. Data were deliberately collected from a variety of sources, namely staff and consumers with varying demographic characteristics, geographic locations and services provided at sites (primarily residential versus outreach). This increased transferability of the research findings. The dependability of the research findings was enhanced by involving two researchers in the data collection and coding. A preliminary report of the research findings was presented to a panel of research academics, consumer experts and community mental health sector workers to ascertain further feedback and refine themes.

## Results

### Recruitment and Demographic Survey Results

Three sites from two organisations provided consent to participate (see **Table 1**). Two sites provided outreach support for consumers. One site provided 24-h residential support to consumers.

**Table 1:** Focus group site, location and participant numbers.

	Location	Consumers (*n* = 17)	Staff (*n* = 31)
**Organisation 1**			
Site 1A (Outreach support)	Regional	5	7
Site 1B (Residential support)	Regional	5	12
**Organisation 2**			
Site 2A (Outreach support)	Metropolitan	7	12

**Table 2** provides the results of the short demographic surveys completed by staff and consumers at the beginning of each focus group. Many consumer participants had a current diagnosis of schizophrenia (47%) or depression (47%). The majority of staff were mental health support workers. Aboriginal or Torres Strait Islander people were over-represented as consumer participants; however, no staff participants identified as Aboriginal or Torres Strait Islander peoples.

**Table 2:** Results of demographic surveys for consumers and staff.

Demographic information	Staff (*n* = 31)	Consumers (*n* = 16)^a^
**Age range**		
16–25	3 (9%)	2 (13%)
26–45	16 (52%)	6 (38%)
46–65	12 (38%)	8 (50%)
Gender^b^		
Female	23 (74%)	7 (41%)
Male	8 (25%)	8 (47%)
**Aboriginal and/or Torres Strait Islander status**		
Yes	0 (0%)	3 (19%)
No	29 (93%)	13 (81%)
Smoking status^c^		
Current smoker	7 (23%)	14 (86%)
Ex-smoker	8 (26%)	2 (13%)
Non-smoker	14 (45%)	0
**Role of health professional**		
Mental health support worker	17 (55%)	−
Other^d^	6 (19%)	−
Peer worker	4 (13%)	−
Manager	3 (10%)	−
Team leader	1 (3%)	−
**Current mental health diagnosis** **^e^**		
Depression	−	8 (50%)
Schizophrenia	−	8(50%)
Anxiety	−	4 (25%)
Bipolar disorder	−	3 (19%)
Schizoaffective disorder	−	3 (19%)
Personality disorder	−	1 (6%)
Other^f^	−	2 (13%)

a While 17 consumers participated in the focus groups, 16 completed the demographic survey.

b Gender missing for one consumer participant

c Smoking status missing for one consumer participant.

d ‘Other’ includes students on work placement, Health Promotion Officers and participants who preferred not to answer.

e Mental illness diagnosis missing for one consumer participant, consumers could tick more than one mental health disorder when responding.

f ‘Other’ includes post-traumatic stress disorder and one consumer participant who preferred not to answer.

### Smoking as an Integral Part of Living and Social Environments

Most consumers reported long histories of smoking, starting when they were in their early teenage years. Consumers identified factors in the living and social environments they grew up in as influential in their initiation of smoking. Living with a parent or carer who smoked, being surrounded by other peers who smoked or working in industries where smoking was the norm was common.

“I was brought up in a foster home where everyone smoked, and it was chronic.”

–Consumer participant (male, occasional smoker, aged 46–55)

“I worked in hospitality industry for 9 years, and it was like a smoke-filled environment anyway. Smoking back then, you walked into the club, and you walked into the smoke.”

–Consumer participant (male, daily smoker, aged 46–55)

“I used to like it as a kid. My old man used to smoke cigars, smoke at least one a day, big fat cigars, and I used to love the smell of it. And then when I was probably in year 6, I started pinching my mother’s cigarettes; she used to smoke Winfield menthol cigarettes.”

–Consumer participant (male, daily smoker, aged 46–55)

The culture of smoking, which included sharing cigarettes and use of smoking to socialise and maintain social networks, was discussed as a significant barrier for quitting smoking and addressing tobacco in general. Opportunities for socialising were limited to being with peers who also smoked. Consumers also talked about the high prevalence of smoking in their communities.

“I think the majority of people smoke. Everywhere you go, there are people in front of you smoking.”

–Consumer participant (female, daily smoker, aged 46–55)

“All my friends smoke, and my mum smokes heaps. Most of my family smoke.”

–Consumer participant (female, daily smoker, aged 18–25)

Staff talked about the historical effects of institutionalisation continuing to have an impact in the present day, particularly for older consumers who may have spent extended periods of time in institutions. Staff criticised systemic issues that led to non-smokers beginning to smoke to access perceived benefits of smoking, for example, short-term leave from inpatient settings.

“Many of these people [consumers] have come out of the local mental health unit or an institution and have had long periods of time, years, in those places. And there is a real culture around smoking in these places; there’s a bartering culture, so some of this stuff is entrenched.”

–Staff participant (female, ex-smoker, aged 56–65)

“[Consumers will] attend mental health–specific groups, and all of them go and stand in the back garden and smoke together. I met someone who was admitted to rehab, and doctors give 15-minute leave, so what else are they supposed to do? Consumers actually picked up smoking just so they could get that 15 minutes’ leave; then they come out, and they make friends in rehab, and they all smoke, and it becomes a habit and a social thing.”

–Staff participant (male, ex-smoker, aged 46–55)

### Smoke-Free Living Environments

The majority of consumers were living in a unit, bedsit or apartment in an apartment complex. One consumer talked about living in a house. Most lived alone, some were living with co-tenants, and some consumers had children that visited and stayed with them periodically. A number of consumers talked about neighbours in nearby apartments who smoked. Smoking in the home was a common and normal occurrence for almost all consumers.

“You’re not allowed to smoke inside, but I do … In the kitchen, I smoke bumpers. I don’t smoke a full cigarette inside, just a little short one.”

–Consumer participant (female, daily smoker, aged 46–55)

“…when I’m there on my own, I smoke anywhere I want in the house.”

–Consumer participant (male, daily smoker, aged 46–55)

Reasons for smoking inside the home included practical considerations such as hot or cold weather, lack of balconies, neighbours asking for cigarettes, unsafe neighbourhoods, living alone and social isolation. Consumers and staff highlighted smoking in the home as an act of consumers maintaining control and sense of choice in lives where there was a limited sense of choice and control.

“…there’s not many places you can smoke anymore; you’re sort of limited. There’s lots of places that people would like to smoke in, but they’re not allowed to.”

–Consumer participant (female, daily smoker, aged 46–55)

“The isolation, living alone, it’s their space; they’re not impacting on anyone else but themselves in that space.”

–Staff participant (female, daily smoker, aged 36–45)

“People don’t have a lot of control in their life, so in their own home, they choose to smoke inside because that’s their choice.”

–Staff participant (female, daily smoker, aged 26–35)

A small number of consumers talked about enforcing their own ‘no smoking’ rules inside the home because they did not like the smell of smoke in the home, they had previously quit smoking or they had children and were worried about the impact on their health.

“If you’re on your own, I think it’s acceptable. But if you’ve got kids and you’re smoking in front of them, and then they’re inhaling the toxins.”

–Consumer participant (female, daily smoker, aged 26–35)

Staff confirmed that smoking inside the home was a regular and normal occurrence for consumers and expressed fatalistic views regarding the utility of attempting to address smoking in consumers’ living environments.

“They’re going to smoke when we’re not there. They’re not supposed to smoke in their property, but they do. You can’t stop it, but you could discourage it.”

–Staff participant (male, ex-smoker, aged 46–55)

“They’re going to smoke anyway; better to be open about it, and if we put that boundary, there then we’ll never have that opportunity to have those conversations.”

–Staff participant (male, ex-smoker, aged 36–55)

The physical design of apartment complexes was also discussed by staff who felt that the configuration of complexes could either enable or inhibit consumers to implement smoke-free homes. Shared common areas in complexes tended to be designated smoking areas (either formally or informally), particularly for people who lived on their own. Complexes with less shared common space were seen as promoting smoke-free homes by discouraging socialising. The design of units and proximity of neighbours who smoked was also raised as a contributing factor to increased second-hand smoke exposure.

“The two [consumers] that keep on smoking, they share a wall, and so they talk over the wall, and they can smell the smoke.”

–Staff participant (male, ex-smoker, aged 46–55)

“…the configuration of the units was slightly different … it was slightly less social in those properties. There was no common area; other properties do have a common area. I think that could help.”

–Staff participant (female, daily smoker, aged 36–55)

However, there was unanimous agreement across all participants that passive smoking was a risk to health.

“Passive smoking can make you ill. When I was a non-smoker and my friend used to smoke, and when I breathed it in, I had to go on antibiotics.”

–Consumer participant (female, daily smoker, aged 46–55)

“If consumers want to have a smoke, I let them know I don’t want to be part of it; I’ll be over here away from them.”

–Staff participant (male, non-smoker, aged 46–55)

### Smoke-Free Environments and Consumer Engagement

All staff expressed positive views on the potential role of community mental health organisations to help smokers quit. Staff who were smokers were primarily concerned about their responsibility to be positive role models for consumers. Staff who smoked described strategies they used to ensure that consumers did not see them smoking and even to avoid smelling of cigarette smoke. Many staff who were smokers preferred that consumers were not even aware that they were smokers because they felt hypocritical and disingenuous.

“I would never smoke in front of someone I support; I don’t like them to know I smoke. I always try to mask the smell. If I have one at work, I go far away so no one can see.”

–Staff participant (female, daily smoker, aged 26–35)

Promoting or implementing smoke-free organisations was a conflicting issue for most staff. All staff expressed recognition and concern about the financial, health and social impacts of smoking. Staff understood why consumers smoked and the impact of consumers’ wider social and living environments on the difficulty of quitting smoking. Yet most staff talked about feeling ambivalent about implementing smoke-free areas, services and homes managed by their organisations. The greatest concern for staff was related to consumers not accessing support if services were smoke-free. Arguments for making services smoke-free were weighed up against the potential for consumers to cease accessing support and risk becoming more socially isolated. Some staff viewed current designated smoking areas as problematic but felt reluctant to remove those areas because they were perceived as often the only opportunities for consumers to socialise and leave the home. Reconfiguring the design of designated smoking areas was raised as a possible compromise by some staff. Current smoking areas were perceived as areas that promoted socialising. Staff suggested making designated smoking areas less inviting so that consumers would be less inclined to remain in the area.

“…if we were to say that you can’t smoke here anymore, I think a significant amount of people would not come [to the community mental health service].”

–Staff participant (female, daily smoker, aged 36–45)

### Tensions Between Cessation Support and Models of Care

Underpinning the ambivalence of staff for smoke-free environments and homes was the conflict with recovery-oriented support, that it was the antithesis of autonomy and undermined self-efficacy.

“I think a blanket rule to say you can’t smoke wouldn’t work for this setting. It wouldn’t fit in with our recovery focus. Our role would need to be recovery focus that gives consumers choice.”

–Staff participant (female, occasional smoker, aged 26–35)

Staff expressed reluctance to provide smoking cessation support to consumers who had not requested it due to the focus in recovery-oriented models of care on choice. Staff emphasised that their role was to provide reactive support to consumers who requested help regarding their smoking, rather than provide proactive support. Staff felt that it was the role of community mental health organisations to support smokers to quit, but this needed to be done in line with recovery and goal-oriented support that focussed on consumer goals and choices.

“…make it very clear that it’s their choice. That’s part of our role; it’s not our place to tell people what they should and shouldn’t do. It’s about supporting their decisions even if we don’t think it might be the best thing. Independence and autonomy.”

–Staff participant (female, daily smoker, aged 26–35)

### Lack of Support and Attitudes of Other People

Other people’s unsupportive attitudes towards quitting smoking and an overall lack of social support were raised as barriers to quitting smoking by consumers. Some consumers talked about a lack of support in relation to being isolated or disconnected from family or other networks. Other consumers with more social support had largely negative views about the prospect of telling someone in their network that they were thinking about quitting smoking. Similarly, staff felt that involving friends and family in a quit attempt may not be a helpful strategy or could even be an impediment to quitting smoking for some consumers.

“…they’ve just laughed at me; it was kind of like “ye sure”, and then you just think, well … what’s the point? You aren’t supporting me in any way, so forget it.”

–Consumer participant (female, daily smoker, aged 46–55)

“…sometimes family just can’t cope with supporting that person, and that causes tension and trauma and pain. So then in those situations, if you don’t feel like you’re supported by your family, why would you ask for help?”

–Staff participant (female, daily smoker, aged 26–35)

### Social Isolation and Exclusion

Across the staff and consumer focus groups, boredom, isolation and loneliness were raised as critical barriers to quitting smoking. Consumers used smoking as a form of company or socialising and as a recreational activity to pass the time in lieu of any other distractions or activities.

“I’m quite isolated where I live, so I tend to, if I’m feeling stressed from the isolation, I’ll smoke for the company of the smoke…”

–Consumer participant (male, occasional smoker, aged 46–55)

“Loneliness is one kind of factor. They’re just really lonely. I asked one of my consumers, “how can you afford this amount of money in the week to spend on smoking?” And he said, “this is my friend; I talk to him while I’m smoking”. He is cut out from the world; he has no family contact, limited friends, so he’s saying this from his heart. “When I light this, it brightens me up.’”

–Staff participant (male, non-smoker, aged 36–45)

“Social inclusion and lack of social participation that the majority of our consumers have. We’re all sitting at work today, and we’re not having a cigarette because we have to be in this room and office, so we can’t. But when you’re in your home and if there isn’t a barrier, apart from whether you can afford to have a cigarette, you can just chain-smoke all day long.”

–Staff participant (female, non-smoker, aged 18–25)

## Discussion

This study aimed to explore the factors within consumer’s social networks and living environments that facilitated or inhibited smoking cessation from the perspective of staff and consumers with SMI. Both staff and consumers discussed the pivotal role that living and social environments play in tobacco use and cessation by people with SMI. Consumers identified smoking as an integral part of life and identified a lack of social support, isolation and loneliness as key barriers to quitting. While many consumers reported smoking inside the home, others described enforcing smoke-free rules. Staff spoke about tobacco use with a degree of fatalism, conceptualised tobacco use as a choice rather than an addiction and highlighted tensions between cessation support and broader models of care. Staff viewed smoke-free home and mental health service policies as effective at promoting quitting but contradictory to recovery-oriented models of care.

Social isolation (including alienation, stigma and loneliness) is commonly reported by people with SMI (35) and is a barrier to quitting smoking (36, 37). Evidence suggests that smoking behaviour is influenced by social networks and that groups of people quit together *via* social contagion (38). Quitting smoking in and of itself may expand a person’s social environment (39). Effective interventions for enhancing social networks exist (40) and can involve guided peer support groups focussing on enhancing social relationships (41) and cognitive and social skills training (42). There is also potential for incentives-based programs paired with peer support to improve social functioning (43). Such programs could address the barrier of social isolation by promoting the positive effects of strengths-based social support in tandem with offering smoking cessation support. The use of peer support to deliver tobacco cessation programs also has potential to overcome social isolation (44). Further research is required to establish the effectiveness of addressing social isolation and use of peer-delivered interventions as part of tobacco cessation programs. Use of tobacco to self-medicate and cope with stress has been identified as a barrier to quitting by people with SMI. In a sample of smokers with schizophrenia, 60% reported smoking to relieve stress and 31% to alleviate symptoms of anxiety and depression (45). Additionally, the tobacco industry has also funded internal and external research to support the self-medication hypothesis (9, 12). However, this did not arise as a key theme in this study. This is most likely due to the focus of the discussion guides, which looked at the factors specifically within a person’s living and social environments.

People with SMI are less likely to live in smoke-free homes than people without SMI (46), and many consumers in the current study were breaching their tenancy agreements by smoking inside the home. One Australian study found that only 31.5% of people with SMI lived in a smoke-free home (46). On a population level, smoke-free homes are associated with increased smoking cessation and decreased cigarette consumption in adult smokers (47). Existing programs are effective at decreasing exposure to second-hand smoke within homes (48). Reflecting the existing literature (49, 50), the factors that facilitated smoke-free homes in the current study included presence of children and those with health issues that are exacerbated by tobacco smoke, concern over effects of second-hand smoke, suitable designated smoking areas, safe neighbourhoods and not liking the smell of smoke in the home. Further research is required to examine effective interventions for promoting smoke-free homes for people with SMI. Tenants and public and private stakeholders should be involved in developing, implementing and evaluating smoke-free home policy (51).

Fatalism that tobacco use is inevitable and that quit attempts will fail has been documented in previous studies exploring staff attitudes to addressing tobacco (52). Evidence suggests that fatalistic beliefs may serve several functions including to save face, to manage uncertainty, to relieve stress or to make sense of current experiences (53). In the current context, staff fatalism may be a response to the perceived complexity of addressing tobacco and a sense of powerlessness to affect change. Between 2.5% and 55.1% of mental health professionals believe that smoking cessation interventions are ineffective (15). Even trained smoking cessation counsellors in the UK Stop Smoking Services identify a need for further training to support people with mental illness, with 77.4% of counsellors wanting more training on the effects of quitting smoking on mental health (54). It is especially important to address staff fatalism given the association between consumers’ *perceptions* of staff support and quitting. Higher levels of perceived staff support have been associated with a greater number of quit attempts (55). Opportunities to increase staff optimism about the possibility of consumers quitting smoking including training for staff and targeted marketing campaigns to the community mental health sector may help to address fatalistic attitudes.

Staff consistently upheld the view that tobacco use was a consumer’s choice and that reactive rather than proactive support should be offered. This is supported by a meta-analysis of mental health professionals’ attitudes that found that 51.4% (pooled proportion) of staff felt that people with SMI were not interested in quitting smoking (15). This is despite evidence indicating that people with mental illness are just as motivated to quit smoking as those in the general population. A meta-analysis found aggregated data from nine studies that indicated that more than 50% of smokers with mental illness are planning to quit within the next 30 days to six months (20). Conceptualising smoking as a choice is problematic as it ignores the physiological addiction caused by nicotine (56), the young average age of smoking initiation (57) and the social determinants of tobacco use and health (58). Additionally, the tobacco industry uses the argument of choice to shift the responsibility of the harms of tobacco to smokers and to minimise the powerful role the industry has in shaping individuals’ environments in ways that are detrimental to individuals’ health (59).

Tensions between recovery-oriented models of care and addiction treatment have been documented (60). The tension described by staff between addressing tobacco and recovery-oriented models of care deserves further discussion. Staff were concerned that smoke-free environments were antithetical to the principles of recovery-oriented models of care that emphasise autonomy, independence and consumer-driven goals. Viewing tobacco use as a choice and low confidence in the efficacy of staff-delivered support were interlinked with this perspective. However, this perspective does not acknowledge the contribution of quitting smoking to recovery in mental health including reducing stress and increasing quality of life (22). Furthermore, staff rarely referenced consumers’ goals or preferences in receiving support for smoke-free environments or cessation or factors within social and living environments that may prevent consumers from making informed decisions about cessation. The extent of this tension between recovery-oriented models of care and provision of other preventive health support or advice is unknown. It is possible that staff also experience a tension when required to address other behaviours, for example, illicit drug use, nutrition, physical activity, alcohol and sexual health. This has implications for the broader aim of addressing the physical health needs of people experiencing mental illness. In acknowledging the broader influence of living and social environments, creating smoke-free environments and addressing nicotine dependence enable people with SMI to exercise autonomy in considering alternatives to smoking. Further research is required to ascertain how addressing tobacco may be conceptualised as part of recovery models of care from the perspective of consumers, staff and carers.

Pooled proportions from the published literature indicate that mental health professionals report lack of knowledge, training and skills (35.8%) and low confidence (31%) as barriers to supporting people with mental illness to quit (15). The published literature and the results of the current study highlight the importance of continuing to provide education and training to community mental health staff that people with mental illness are interested in quitting and are capable of quitting smoking and that quitting smoking positively impacts on mental health and quality of life and supports recovery (9). Training could also address the physiological effects of nicotine, tobacco industry interference and the broader social determinants of health and how these influences might curtail the ability of a person to make informed choices. There is the potential to review learning curricula in key tertiary courses at both universities and technical colleges to ensure that people entering these professions understand these concepts early on in their professional careers. Training to address these myths and impart smoking cessation support skills needs to form part of broader, organisation-wide interventions. Organisational or systems change interventions require a multifaceted approach, involving multidisciplinary and ‘multi-level’ collaboration from senior management, staff, consumers and carers to develop, implement and evaluate policies, procedures and processes that support the routine and consistent addressing of tobacco. Organisational interventions are effective at changing practice within healthcare settings (61) and have potential to be effective in mental health services settings (62).

Community mental health organisations are well placed to address issues such as social inclusion and smoke-free homes as part of their provision of psychosocial care (6, 63). A sample of community service sector managers surveyed found that 86% felt positively about providing support and encouragement to quit to their clients (64). However, community mental health organisations will require additional resourcing and support to do so. These findings indicate that engagement with a broader range of key stakeholders will be required to address tobacco within the living and social environments of people with SMI, e.g., housing, employment, planning and development and all levels of government. It is not the intention of this research to reflect negatively on the work of staff or their perceptions of smoking and mental health consumers. Staff clearly articulated knowledge on the negative impacts of smoking and even the broader social and economic factors that drive smoking rates in populations such as people with SMI. The aim of this work was to highlight areas where staff require further support to continue the important work they do in providing care for consumers. It is equally important to recognise the scope and boundaries of the work done by community mental health staff. Issues such as appropriate smoking cessation pharmacotherapy, medication interactions and monitoring of withdrawal symptoms require input and collaboration with those who provide clinical care. Carers also play a role in advocating for the provision of smoking cessation support by staff (65) and supporting cessation for people with SMI (55).

### Strengths and Limitations

The inclusion of services that provide psychosocial support to people with SMI including primarily psychotic disorders is a strength of this study. Additionally, the sampling frame allowed the inclusion of services that provided outreach or residential support, ensuring participation by consumers with varying levels of support needs. The findings of this study may reflect the social and living environment impacts of other priority populations without SMI, e.g., people seeking treatment for drug and alcohol problems and people from more disadvantaged socioeconomic backgrounds. However, the results of this study should be interpreted considering a number of study limitations. The results of this study may not be transferable to other mental health services (e.g., inpatient or private) and may not reflect experiences within rural communities. It is possible that there may have been key differences between consumers who decided to participate and those that did not, and these findings may not generalise beyond those who took part in the study. However, generalisability is not an aim of qualitative enquiry. Rather, the aim is to gather rich and detailed data from a specific sample.

Key future recommendations arising from this paper include:

- Examining the effectiveness of tobacco cessation interventions that include components to improve social inclusion, social support and organisational change within community mental health organisations
- Utilising population- and targeted-based interventions, adapted for mental health populations, to enhance awareness and implementation of smoke-free environments, including smoke-free homes
- Exploring the impact that resourcing has on community mental health organisations’ ability to provide routine and comprehensive smoking cessation support
- Exploring staff, consumer and carer perspectives on definitions of recovery and how addressing tobacco can align with these models
- Continuing to build on the work already done with carers and family as support networks to help people with SMI quit smoking
- Ensuring that education and training programs for prospective and current staff in the community mental health sector address the key misconceptions identified in this paper
- Promoting multisectoral partnerships in addressing tobacco including fields other than health, e.g., housing, employment, planning and development and all levels of government

### Conclusions

Consumers identified smoking as an integral part of life and identified a lack of social support, isolation and loneliness as key barriers to quitting within their social networks. While many consumers reported smoking inside the home, others described enforcing smoke-free rules. Staff spoke about tobacco use with a degree of fatalism, conceptualised tobacco use as a choice rather than an addiction and highlighted tensions between cessation support and broader models of care. Staff viewed smoke-free home and mental health service policies as effective at promoting quitting but contradictory to recovery-oriented models of care. There is great potential for the community mental health sector to address tobacco use by consumers through addressing some of the factors within consumers’ living and social environments. However, more education and training to increase staff awareness of the issue coupled with effective programs that target factors within the social and living environment of people with SMI are required. Community mental health organisations are well placed to address many of the factors within consumers’ living and social environments; however, they must be properly resourced to do so. Multisectoral involvement in addressing tobacco is required at the level of living and social environments.

## Tackling Tobacco Mental Health Advisory Group

Karina Ko, Centre for Population Health, NSW Ministry of Health, North Sydney, NSW, Australia; Christopher Oldmeadow, Clinical Research Design, IT, and Statistical Support (CReDITSS), Hunter Medical Research Institute, Wallsend, NSW, Australia; Sharon Lawn, Flinders Human Behaviour and Health Research Unit, Department of Psychiatry, Flinders University, Adelaide, SA, Australia; Marianne Weber, Cancer Research Division, Cancer Council NSW, Woolloomooloo, NSW, Australia; Jennifer Bowman, School of Psychology, Faculty of Science and Information Technology, The University of Newcastle, University Drive, Callaghan, NSW, Australia and Hunter Medical Research Institute, Clinical Research Centre, New Lambton Heights, NSW, Australia; Marita Hefler, Tobacco Control Research Program, Wellbeing & Preventable Chronic Diseases Division, Menzies School of Health Research, Casuarina, NT, Australia; Philippa Boss, Sydney Medical School, University of Sydney, Camperdown, NSW, Australia.

## Ethics Statement

This study was carried out in accordance with the recommendations of the National Statement on Ethical Conduct in Human Research. The Cancer Council NSW Human Research Ethics Committee (#306) approved the study protocol and all subjects gave written informed consent in accordance with the Declaration of Helsinki.

## Author Contributions

LT, SCW, ALB and BB conceived and designed the analysis. CC collected the data and performed the analysis. LT and CC wrote the initial draft of the paper. All authors including the Advisory Group were involved in reviewing draft manuscripts and contributing to interpretation of results. All authors read and approved the submitted version.

## Funding

This study was funded by the NSW Ministry of Health.

## Conflict of Interest Statement

The authors declare that the research was conducted in the absence of any commercial or financial relationships that could be construed as a potential conflict of interest.
